# Muscle strengthening intervention for boys with haemophilia: Developing and evaluating a best‐practice exercise programme with boys, families and health‐care professionals

**DOI:** 10.1111/hex.13119

**Published:** 2020-08-17

**Authors:** Ferhana Hashem, David Stephensen, Wendy I. Drechsler, Melanie Bladen, Liz Carroll, Pellatt‐Higgins Tracy, Eirini‐Christina Saloniki

**Affiliations:** ^1^ University of Kent Centre for Health Service Studies Canterbury UK; ^2^ East Kent Hospitals University NHS Trust Haemophilia and Thrombosis Centre Canterbury UK; ^3^ Royal London Hospital Haemophilia Centre London UK; ^4^ Kings College London School of Population Health & Environmental Sciences London UK; ^5^ Great Ormond Street Hospital For Children NHS Foundation Trust Haemophilia Centre London UK; ^6^ Institute of Child Health University College London London UK; ^7^ Haemophilia Society UK London UK; ^8^ University of Kent Personal Social Services Research Unit Canterbury UK

**Keywords:** Boys, Exercise, Haemophilia, Life‐experience, Muscle strength, Patient adherence, Physiotherapy

## Abstract

**Background:**

Muscle strengthening exercises have the potential to improve outcomes for boys with haemophilia, but it is unclear what types of exercise might be of benefit. We elicited the views of health‐care professionals, boys and their families to create and assess a home‐based muscle strengthening programme.

**Objective:**

To design and develop a muscle strengthening programme with health‐care professionals aimed at improving musculoskeletal health, and refine the intervention by engaging boys with haemophilia and their families (Study 1). Following delivery, qualitatively evaluate the feasibility and acceptability of the exercise programme with the boys and the study's physiotherapists (Study 2).

**Design:**

A person‐based approach was used for planning and designing the exercise programme, and evaluating it post‐delivery. The following methods were utilized: modified nominal group technique (NGT) with health‐care professionals; focus group with families; exit interviews with boys; and interviews with the study's physiotherapists.

**Results:**

Themes identified to design and develop the intervention included exercises to lower limb and foot, dosage, age accommodating, location, supervision and monitoring and incentivization. Programme refinements were carried out following engagement with the boys and families who commented on: dosage, location, supervision and incentivization. Following delivery, the boys and physiotherapists commented on progression and adaptation, physiotherapist contact, goal‐setting, creating routines and identifying suitable timeframes, and a repeated theme of incentivization.

**Conclusions:**

An exercise intervention was designed and refined through engagement with boys and their families. Boys and physiotherapists involved in the intervention's delivery were consulted who found the exercises to be generally acceptable with some minor refinements necessary.

## 1 INTRODUCTION

Haemophilia is a bleeding disorder associated with bleeding into the muscles and joints.^1^ It is a rare inherited disorder affecting 1:10 000 people where the blood does not clot normally. Over a period of time, repeated joint bleeding leads to chronic synovitis (inflammation of the joint) and arthropathy, which is associated with chronic joint deformity, pain, muscle atrophy and functional impairment. The recommended treatment for people with severe haemophilia is administration of prophylaxis (infusion of the clotting factor concentrate) to prevent bleeding and minimize long‐term arthropathy.^2^, ^3^ Often exercise is used as an aid to recovery after episodes of musculoskeletal bleeding and can help to improve joint function.^4^ Exercise interventions produce improvements in outcome measures including pain, range of motion, strength and walking tolerance.^1^ There is evidence to suggest that exercise for adult males with haemophilia can help with increasing the range of motion and muscle strength enabling rapid mobilization and recovery of function.^1^, ^3^ Yet, there is a lack of robust evidence to determine whether muscle strengthening exercise can improve or negatively affect outcomes for young boys with haemophilia and it is unclear what types of exercises might be of benefit.^1^

Due to the small‐sized patient population and limited validated outcome measures, large randomized controlled trials (RCT) to establish whether an intervention is effective in rare diseases like haemophilia are often difficult to conduct without establishing whether a study is feasible.^5^, ^6^ In addition, resource use may not be optimally rationalized when a treatment or intervention is found to be ineffective or unsafe, or conversely, a treatment or intervention is not provided if it turns out to be effective.^5^, ^7^

Involving patients, carers and health‐care providers at the early stages of intervention development and evaluation is widely recognized as good practice to elicit users’ and practitioners’ views in order to create a credible and motivating programme.^8^ Inductive qualitative methods can contribute to intervention development gaining an in‐depth appreciation of how users may relate to a resulting intervention content and format, and allowing for modifications to take place as necessary.^8^, ^9^, ^10^ McDermott et al^11^ summarize how integral users’ and practitioners’ views are in the development of an intervention, which can help to clarify the mechanisms through which the intervention works, identify potential barriers to change, provide information on individual needs to users and explore relevant issues which can be used to further develop and refine the intervention model.^11^ Qualitatively exploring the acceptability and feasibility of an intervention following delivery is critical to tailor advice and techniques and modify the intervention to make it more usable, relevant, persuasive, accessible and engaging.^9^, ^12^, ^13^, ^14^ Using iterative qualitative approaches can help research teams make modifications to the intervention, as users report parts of the intervention they find hard to perform or indicate problems in reporting their physical activity levels correctly.^13^

The current findings discussed in this paper report upon a recently completed feasibility study funded by the UK’s National Institute for Health Research (NIHR) known as the ‘DOLPHIN’ study or **D**evelopment **O**f a haemophi**L**ia **P**hysiotherapy **IN**tervention for optimum musculoskeletal health (PB‐PG‐0215‐36091).^15^ This paper describes how a muscle strengthening intervention was designed, developed and refined for boys with haemophilia. It involved gaining an in‐depth understanding of the users’ needs and goals making the intervention more relevant, accessible and engaging. A person‐based approach was used in both stages of the study: during the early stages of designing, developing and refining the intervention (Study 1), and then utilized to evaluate the acceptability and feasibility of the intervention (Study 2).^13^, ^16^

## 2 OBJECTIVES

The study was carried out in two phases.

The aims of Study 1 were to engage boys with haemophilia, their families and health‐care professionals to design, develop and refine a best‐practice muscle strengthening exercise intervention aimed at improving musculoskeletal health by:

- Exploring the perspectives of clinicians experienced in paediatric haemophilia care
- Exploring the perspectives of boys with haemophilia and their families

The objective of Study 2 was to qualitatively evaluate the acceptability and feasibility of the exercise programme and re‐visit the intervention to amend any parts before progressing to a final trial by:

- Discussing and capturing feedback with the participants (boys and parents) and the study's physiotherapists to identify any aspects requiring attention to help deliver the intervention for the larger study.

## 3 METHODS

### 3.1 Study design

We used the principles of a ‘person‐based’ approach for intervention development (Study 1) and evaluation (Study 2). The person‐based approach enables intervention developers to understand how different people in different situations may view and engage with the intervention, and identify which elements may be relevant or may be rejected, thus helping to understand how the intervention could be more attractive, persuasive and feasible to implement in a larger study. According to Yardley et al, ^13^ the core elements of a person‐based approach involve (a) intervention planning, (b) intervention design and (c) intervention evaluation of acceptability and feasibility. Due to time constraints, we combined elements (a) and (b) into Study 1 and undertook the evaluation of feasibility and acceptability in Study 2.

In Study 1, the intervention developers (academic physiotherapists, paediatric musculoskeletal physiotherapists, specialist haemophilia physiotherapists and members of the research team) designed the programme using their experiences and knowledge of the user group, as well as key messages presented from a literature review. Following development, the exercise programme was demonstrated to the boys and their families and then refined taking into account their views and actions.^16^

In Study 2, following the delivery of the intervention, an evaluation was carried out to check whether the changes and refinements were successful in making the exercise programme acceptable and interesting and would enable the users to adhere to the intervention in the larger trial.

Qualitative data were collected by an experienced researcher with over 10 years’ experience of data collection with children.

### 3.2 Ethical approval

For Study 1, ethical approval was granted by the University of Kent School of Social Policy, Sociology and Social Research—SRC Research Ethics Committee (SRCEA id 177). For Study 2, ethical approval was granted by the Health Research Authority and London—Fulham Research Ethics Committee (17/LO/2043).

### 3.3 Data collection

Study 1—Designing, developing and refining the best‐practice muscle strengthening intervention.

*Perspectives of health‐care professionals:* Qualitative data were collected from health‐care professionals (n = 11) in January 2017 at the UK Haemophilia Society in London during one modified nominal group technique (NGT) discussion meeting (Table 1). Key findings from a literature review (unpublished) of interventional studies undertaken by DS, WD and MB from January to December 2016 were presented to health‐care professionals in order to stimulate ideas for a prioritization exercise to identify key characteristics for *designing* and *developing* an exercise programme through peer discussion and consultation. The messages from the literature review used to design and develop the intervention included: (a) *strong muscles are important because they help protect your joints from bleeds*; (b) *regular exercise can actually help prevent bleeds and joint damage*; and (c) *exercise can strengthen muscles to support joints*.^1^, ^17^, ^18^, ^19^ The group designed a draft intervention with regard to specific exercises (including frequency, intensity and timing), setting, and length of intervention and training needs.

**Table 1:** Overview of data collection

Data source	Participants	Timeline of data collection	Type of data	Mode of collection
Modified NGT and focus group	**Health‐care professionals** including academic physiotherapists (n = 2), paediatric musculoskeletal physiotherapists (n = 2), specialist haemophilia physiotherapists (n = 7)	January 2017	Qualitative	Semi‐structured discussion collected as focus group data; NGT self‐completion questionnaire using a Likert scale
Focus group	**Families** including children (n = 5), parents (n = 5)	May 2017	Qualitative	Semi‐structured discussion collected as focus group data; Likert scale self‐completion questionnaire
Interview data	**Children** (n = 9), **parents** (n = 9)	October 2018 to April 2019	Qualitative data	Semi‐structured discussion collected via one‐to‐one interview
Interview data	**Study physiotherapists** (n = 2)	March and April 2019	Qualitative data	Semi‐structured discussion collected via one‐to‐one interview

A modified NGT was utilized to facilitate the discussion which was conducted by FH, and involved five stages: introduction and explanation, silent (independent) generation of ideas, sharing of ideas, group discussion, and ranking and voting. After sharing of ideas and discussion, participants were asked to anonymously rate key statements about the exercise intervention on a 4‐point Likert scale (strongly disagree; disagree; agree; and strongly agree).^20^, ^21^, ^22^ This was followed by a focus group discussion.^23^ The modified NGT and focus group discussion were 175 minutes in length (with a short break in the middle). The focus group was audio‐recorded, transcribed and anonymized, which, together with the NGT ratings, informed the design of the draft exercise intervention.

*Perspectives of boys and parents:* Qualitative data were collected from boys and parents (n = 10) at one focus group in May 2017 at the UK Haemophilia Society in London. The draft intervention was demonstrated to the boys and parents. Facilitated by FH, views in regard to suitability of the exercises, increasing adherence and how the exercises could be improved were explored and discussed. The boys and their families were asked to anonymously rate separately the difficulty and the suitability of each exercise on a 4‐point Likert scale (strongly disagree; disagree; agree; and strongly agree). This was followed by a focus group discussion. The focus group discussion was 75 minutes in length (with a short break in the middle). The focus group was audio‐recorded, transcribed and anonymized and together with the Likert scale ratings further informed the design of the exercise intervention.

**Figure 1:**
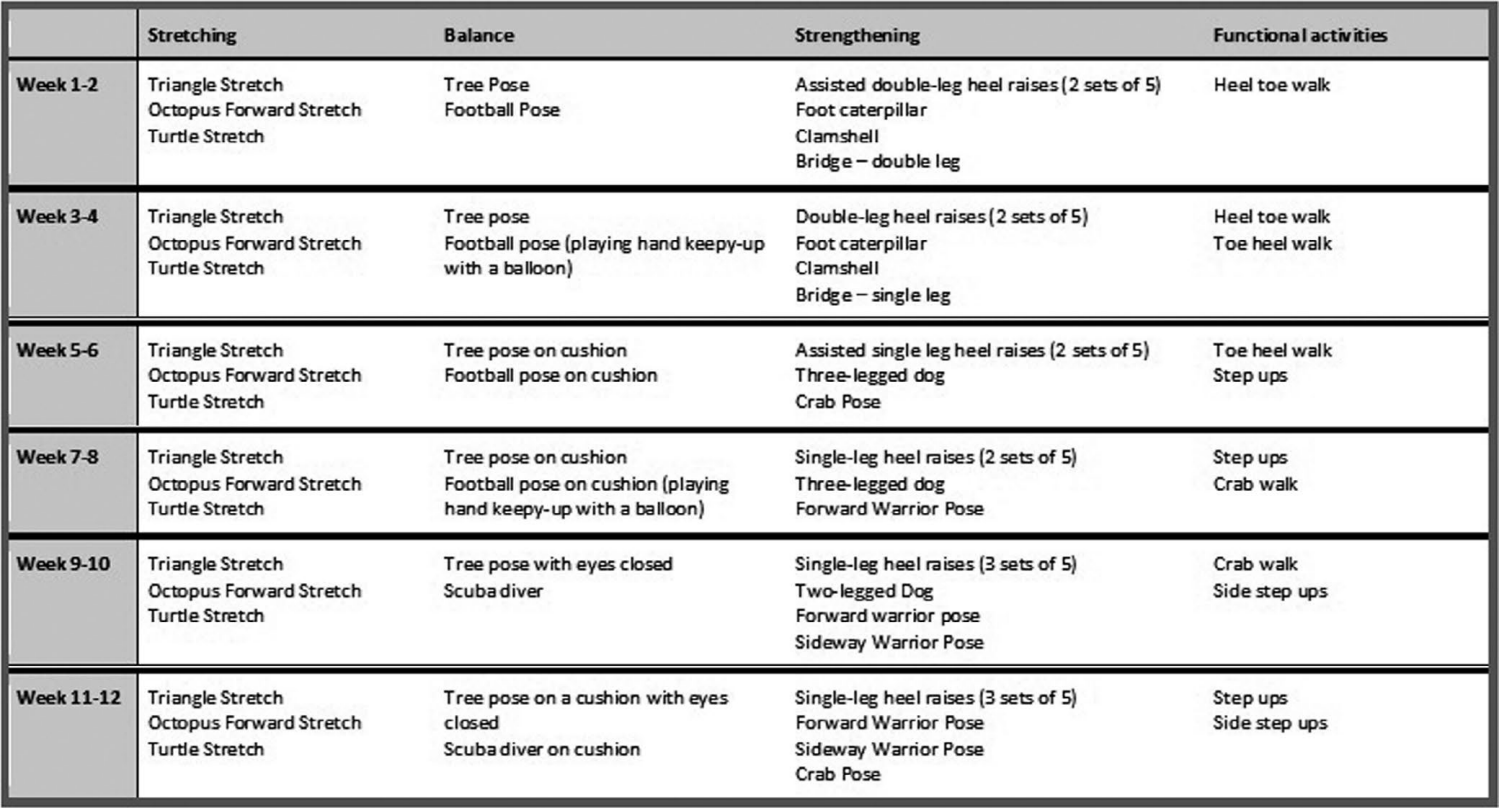
DOLPHIN Exercise programme

A description of the final exercise intervention is provided in Figure 1.

Study 2—Evaluating the best‐practice muscle strengthening intervention for the larger trial.

*Perspectives of health‐care professionals and boys plus parents:* Qualitative data were collected between October 2018 and April 2019 from boys plus parents and physiotherapists. Two data sources were generated: (a) one‐to‐one interview data with boys and parents (n = 18)^24^ and (b) one‐to‐one interview data with the study's physiotherapists (n = 2).^24^ Data collection with the boys plus their parents took place at two case study sites based in hospital trusts in London and the south‐east of England, or with parental permission over the phone from the boys’ homes. The interviews with the families ranged from 6 to 26 minutes. Data were collected and analysed by FH. Data collection with the study's two physiotherapists took place in an informal office setting away from the main two case study sites, and data were collected by FH. The interviews with the study's physiotherapists were 38 minutes and 60 minutes each in length. All interviews were audio‐recorded, transcribed and anonymized.

### 3.4 Participant recruitment

For Study 1, health‐care professionals were recruited through UK haemophilia and physiotherapy networks by the study's principal investigator (DS), who used snowballing techniques to invite interested individuals to take part, which included academic physiotherapists (n = 2), paediatric musculoskeletal physiotherapists (n = 2) and specialist haemophilia physiotherapists (n = 7). Boys and their parents were informed of the study by the UK Haemophilia Society who responded to a recruitment advertisement circulated via a newsletter. Boys were screened for eligibility (inclusion criteria: aged 6‐11 years, severe or moderate haemophilia A or B, with or without inhibitors for prophylactic treatment with coagulation factor, boys with or without symptoms of joint damage). In total, there were five children and five parents. There were four dyads/triads (n = 4): two mothers with a single male child each; one mother with two male children; and a father and mother with a single male child. As with the health‐care professionals, the boys and parents contacted DS directly who obtained informed consent from all participants by way of sending out a participant information sheet and attaining written consent from each participant on each of the data collection days in London.

For Study 2, once boys were screened for eligibility (inclusion criteria: as described above; exclusion criteria: von Willebrand disease, history of fracture or trauma to lower limb, orthopaedic surgery, acquired brain injury or other disturbance of the central nervous system, joint or muscle bleed in lower limb in the past 6 weeks, presence of lower limb pain or unable to fully comply with verbal instructions), the boys and parents were invited to take part in the exercise programme and provided informed consent, which was undertaken as part of the overall feasibility study. In the exercise group, the boys’ ages ranged from 6 to 11 (M = 9.55, SD = 2.79) (n = 5), and in the usual care group, the ages ranged from 8 to 11 (M = 10.00, SD = 1.46) (n = 4). Regarding recruitment of the study's two physiotherapists, they were invited to take part by DS who were sent a participant information sheet with written consent being obtained in advance of the one‐to‐one interviews.

### 3.5 Data analysis

Analysis of the modified NGT data and focus group discussion (Study 1) from the health‐care professionals was twofold. The NGT rating data were entered into an excel spreadsheet, and the number of participants who strongly disagree, disagree, agree and strongly agree with each statement was reported as frequencies and percentages. Analysis of all focus group data in Study 1, one‐to‐one interview data with the boys and parents and the study's two physiotherapists in Study 2, were supported by the use of a qualitative software analysis programme (NVIVO 12 Pro). A thematic approach was used for data analysis.^25^, ^26^

## 4 RESULTS

### 4.1 Study 1—Designing, developing and refining the best‐practice muscle strengthening intervention

#### 4.1.1 Perspectives of health‐care professionals and boys plus parents

**Figure 2:**
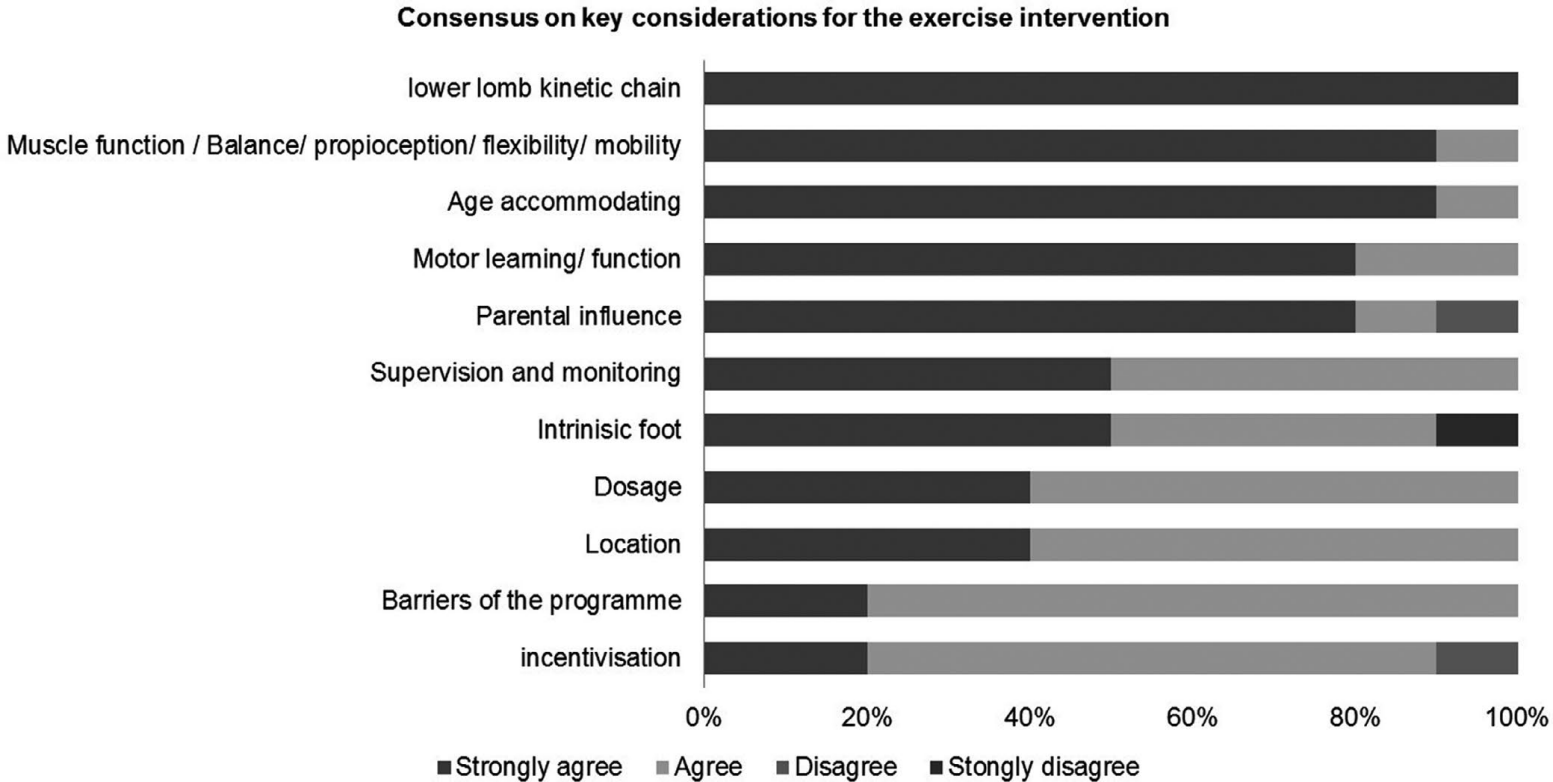
Consensus on key considerations for exercise intervention

The health‐care professionals reached an agreement on what would constitute an appropriate, suitable and practical exercise programme for boys with haemophilia, as well as factors that would influence the delivery of the intervention. The initial ideas were generated and shared by the health‐care professionals to inform the design and development of the intervention and are outlined in Table 2. The Likert scale questionnaire results are given in Figure 2. The five boys who participated in the focus group strongly agreed that they ‘could do’ 14 out of 18 different exercises (78%) from the image and written instructions provided. One of the five families indicated that they needed additional instruction to carry out the remaining four exercises, and the families reached a consensus on alternative instructions for these four exercises that would enable them to understand and carry them out. The boys indicated the need for: pictorial images and guidance on how to perform the exercises; identifying that the home setting was the most appropriate location; completing the exercises within a 30‐minute session was seen as important; and carrying them out twice weekly. Parents considered incentivization would be helpful to encourage adherence. Parents and boys understood that taking part in a trial would involve a computer deciding whether they would receive the intervention or usual care, and they expressed that this would not deter them from being involved.

**Table 2:** Initial ideas generated and shared by health‐care professional in the modified NGT exercise

Characteristics	Type
Motor learning principles	Endurance/strength/flexibility/function
	Goal diverted
	Slow repeated follow‐up variable performance
	Consolidated by sleep
Types	Adherence/enjoyment
	Cost‐benefit analysis
	Principles of specificity
Adjuncts	Mirrors
	Verbal and physical feedback
Setting—enriched environment	Consider daily vs home
	Simple vs complicated programme
	Feedback
	Time of day
Muscle function	Balance high‐level
	Endurance
	Power
	Low impact
	Cardio
	Strength
	Pelvic control work
	Kinetic chain
Ankle	Gait walk
	Turn
	Impact
	Balance
Dosage and frequency	Dependent on motor training type
	Additional to normal activities
	Controlling the effects of other activities
Format—type of programme	Home—reliant on parents
	Gym—formal, learning discipline
	Best case if becomes ‘normal’ life activity
Individualizing any programme	Personality
	Interests
	Baseline fitness measures
Medical lateral control	Load: stepping‐patterns‐pace under/over through games diagnosis
	Levels‐dimensions‐diagnosis
	Stable/instable: ball games, reaching games and tracking games
	Proximal stability mechanism
	Gluts MED/MIN
	Adductor/abductor muscles
Home‐based	Lifestyle
	Going beyond childhood
Parents	Simple guidance for parents
	Reliant on parents
	Incentivization
Balance/proprioception/strength	Age—isometric
	Dynamic strength
	For function
	Altered weights, lunges, squats and weighted squats
Repetitions/time	Determines fatigue
	Needs to be individualized
	Needs explanation
Flexibility	Rigid levels vs mobile adapter
	Flexibility to stretches
Weight bearing vs non‐weight bearing	
Determine dose	Times/repetitions for motivation
Age dependent	
Foot intrinsic	Strength mobility, picking games and dawning games

##### Theme 1: Exercises to lower limb and foot

The clinicians recommended that the intervention needed to include exercises for the lower limb and foot focused on strength, balance, proprioception, flexibility and mobility as these were considered the key impairments related to musculoskeletal bleeding:

…it's about the lower limb and then it's not about the lower limb, it's about trunk control so it's not just ankle…

…I thought maybe something about bare foot, again, with this flexibility intrinsic…I think you just put muscle function. I’d take ‘power’ out because power is one component to muscle function (Health‐care professionals—Modified NGT)

It was also suggested that inspiration for exercises should be sought from gymnastics, yoga and dance training as these activities have a focus on control through range, as well as an emphasis on motor patterns (ie everyday activities).

##### Theme 2: Dosage

The health‐care professionals suggested that the dosage for the exercise intervention would practically and clinically be at around twice a week and comprise a minimum of 16 exercise sessions. The group also agreed that the intervention should be progressive in terms of load and intensity:

I never hear of very many who do an activity five days a week. They usually do their activity maybe once or twice a week, whether it’s their swimming or their football or their something. (Health‐care professional—Modified NGT)

The dosage identified concurred with what was suggested as practical by the parents:

Parent: Twice a week, probably

Parent: If it's just something like half an hour or something like then that's all right (Parents—focus group)

##### Theme 3: Age accommodating

There was strong consensus from the health‐care professionals that the intervention needed to be age‐appropriate to account for neuromuscular maturation and development:

…there are different factors that might be important depending on their age so I think there is more than one way of splitting the age groups which would be a big thing to try and figure out for…You know, if they're growth spurting you might want a massive influence on stretching whereas you don't need that for a six to ten year old or… but with a 6 to 10 year old you might want a huge impact on balance and control…(Health‐care professional—Modified NGT).

It was recommended from the focus group discussion that the age of the participants was limited to a younger age group from age six to 11.

##### Theme 4: location

The focus group with the boys and families helped to identify that the home setting was the appropriate location for delivering the intervention:

Parent:…in the living room to do it quite easily I think so. If you set up a YouTube thing or like a DVD they’ve got something they can just turn on then follow…and then when they’re done they’re done. I know it sounds silly. Kids do follow technology like that these days. (Parent—focus group).

The health‐care professionals also considered where the most suitable setting would be, with their view concurring with those of the parents, who identified the home as a potential location, outside of a formalized clinical setting:

I was trying to think what do you think would be the best setting for them and therefore can they do it somewhere but they mustn't be made to feel different or special from their peers So I don't know if a school can do it or at home because it's private (Health‐care professional—Modified NGT)

##### Theme 5: Supervision and monitoring

The parents noted that the boys would need to be guided with help for learning the exercises especially at the beginning:

Parent: Also I think, I know because with the angle it’s a bit strange so they need guidance for the beginning. (Parent—focus group)

In addition, the health‐care professionals discussed that the parents’ input to facilitate the exercises to be carried out would need to be moderated. In order to reduce the influence of parental bias and ensure the exercises were completed correctly, the health‐care professionals recommended supervision by a physiotherapist:

…but I also think that also they [parents] will need more support… you've got six weeks of doing these exercises every day with a child who doesn't want to do them in addition to what you’ve got to do in the home environment and I’m just thinking…they [physiotherapist] comes to the house and give you support (Health‐care professional—Modified NGT)

##### Theme 6: Incentivization

The parents discussed the issue of incentivization and indicated it would be helpful to reward the boys through vouchers to keep them interested in a programme that lasted for a number of weeks:

Parent: Sometimes a voucher does something…something like FIFA vouchers, you know all kids are going to be different I know (Parent—focus group)

Incentivization was also considered important to maintain adherence by the health‐care professionals:

There's got to be a little bit of fun so there has to be some humour in it so…because children love the excitement of change or [doing somethings] different or a new toy or a trip or something (Health‐care professional—Modified NGT)

### 4.2 Study 2—Revising the best‐practice muscle strengthening intervention for the larger trial

Physiotherapists, parents and boys reported that the majority of exercises were appropriate, indicating that the way they were structured, including a combination of stretching and body resistance, was suitable. They felt that the exercises were achievable. It was suggested that parents could be supported and trained to help with the intervention by using an online learning or video resource of the exercises. Exercises ‘linked to everyday activities undertaken in the playground such as football proved to be popular and held their interest’. Parents reported that the boys were less compliant with the three stretching exercises at the beginning of each session, which they described as ‘continual and repetitive’.

#### 4.2.1 Perspectives of boys, parents and physiotherapists following intervention

##### Theme 1: Progression and adaptation

The boys and their parents commented about different aspects of the muscle strengthening exercise indicating what was achievable. The interviewer asked why the participant did not like the triangle stretch, and the child stated that:

Child: Well just hard to keep my back straight for when I did it

Mother: It was. But you…towards the end it was easier wasn't it, so… (Parent and child—Study Site 1)

Another parent and child explained how the intervention enabled progression:

Father: …When [name] was talking to you, you know the normal stretches on the floor, when you're sort of bending forward you started off with your hands on your knees almost and then you could get them down past your knees and then when you were standing up doing the stretches you started off finger tips and then where did you end up…?

Child: Palms

Father: Almost your palms, yeah, so you'd gained all that extra and [name] was saying you'd done well doing that didn't he (Parent and child—Study Site 2)

There were challenges identified by parents and physiotherapists around the progression of one of the balance exercises, the ‘scuba dive pose’, where the boys had to maintain a one‐legged position whilst performing dynamic upper body movement. Diary records showed that the boys were able to master this exercise over the course of the programme. The physiotherapists indicated that an adapted version of the scuba diver pose or an additional pose in between the football and scuba diver pose to bridge the gap between the two exercises was worth inclusion for the larger trial.

Physiotherapists: I think the main challenge was the “Scuba pose” really, and it wasn’t…because they only did it for two weeks for the last two weeks. I’m not sure by adding that level of exercise…because it isn’t a progression from, what was the one before… (Physiotherapist—Study Site 2)

##### Theme 2: Maintaining adherence to the intervention

The boys and parents noted that having regular visits (once per week) from the physiotherapist provided a moral boost and helped the boys’ compliance with carrying out the new exercises, as well as practising older ones:

Child: Like when [name] like because when I was doing the exercise in my house [name] would come over like every week. If I was like struggling with one because there was like new ones sometimes she'd like help me out with the new ones like because she came every Monday and gave me a new booklet every two weeks. So when I was on a new booklet she'd help me go over the new ones so then I’d get an understanding of like what they are. (Child—Study Site 1)

The physiotherapists also identified having face‐to‐face contact with the boys in their homes also increased adherence:

Physiotherapist: But actually it's really really worth while…yeah, from the therapist and patient rapport but also from patient compliance but also you can see the difference in that child over a twelve week period, you know, so I would definitely as something we should look at like in the haemophiliac, I know we really don't have the capacity, but it's something…[having] face to face [contact], in their own environment… (Physiotherapist—Study Site 1)

The parents identified that in order to facilitate their child's adherence with the intervention, the exercises were realistic and attainable in a day:

Father: You had a few strops didn't you? You did end up actually doing them didn't you? Even if we split it down…did you find it easier when we split five exercises a day? (Parent and child—Study Site 2)

This father also commented about how important it was to incorporate the exercises into regular time points in the day:

Father: Once we got into the routine it was easy wasn't it. When we got into a routine of having dinner, you going up stairs to play for half an hour or something and then coming back downstairs it was all right wasn't it? (Parent and child—Study Site 2)

One parent and child pair indicated that starting the intervention at the end of the summer in September when temperatures had decreased, meant that the exercises were achievable given the climate, plus the child has the time to undertake the exercises:

Child: Like in the summer I would go out and play football but like as it's winter I don't really go out that much so I had plenty of time to do that

Child: [It was in] September…So it wasn't that hot so I could just…I had plenty of time

##### Theme 3: Incentivization

It is interesting to note that the health‐care professionals and parents in Study 1 argued that having an incentive would increase a child's chances of taking part and complying with the exercise programme. However, interviews conducted following the intervention suggest that this might not be always the case:

Mother: …but I mean we didn't know about the gift card until a couple of weeks after… (Parent—Study Site 1)

Father: We said there may be a treat. But we haven't told him what. It was just a little incentive half way through when he started getting a bit…if you carry on then there might be an incentive in there for you. There might be a little reward at the end. He still doesn't know what the reward may be. (Parent—Study Site 2)

Although the parents felt that having a voucher did not primarily act as an incentive to ensure adherence, one of the physiotherapists noted in fact the parents retained control and accumulated the incentives for the younger boys. Yet for the older boys, they assumed control of expenditures for the voucher and had already planned how to spend it (Physiotherapist—Study Site 1).

##### Theme 4: Identifying training needs of physiotherapists and parents

The physiotherapists commented on the potential training requirements for a future larger RCT. One of the physiotherapists identified the additional training needs for new physiotherapists who had no experience of treating boys with haemophilia:

Physiotherapist: …maybe one face to face session with other physios who could then deliver the intervention…haemophilia’s quite a rare disease and other physios may not have ever come across haemophilia, so you know some training around haemophilia I think would also be necessary because of their needs in some ways being different and around bleeds and things like that so…yeah, because there are only a small number of us that are specifically to haemophilia I think it would have to be training other paediatric physios… (Physiotherapist—Study Site 1)

The other physiotherapist focused on discussing how parents can be supported and trained to help with the intervention by using an online learning resource:

Physiotherapist: I think on‐line tools definitely. More for when…the therapist is not there. If they're in the intervention group and the therapist is not there and you've said to them well adapt it this was or something like this something there for the parents just to go online and have a look this is how the exercise is actually done and then be like this is what we need to copy (Physiotherapist—Study Site 2)

## 5 DISCUSSION

Where physiotherapy management and exercise are being trialled in rare conditions such as haemophilia, it is important to design, develop and refine the components of an intervention using a targeted approach focusing upon the experiences of the users in order to optimize acceptability and feasibility.^27^, ^28^ We have (a) described how a person‐based approach has helped to engage boys, families and health‐care professionals in designing, developing and refining an intervention, and (b) presented evidence to ensure the muscle strengthening exercise programme had the potential to be suitable and appropriate for improving adherence when progressing to a larger trial.

The health‐care professionals involved in our study identified functional movement competencies that the exercise intervention should include, provided advice on suggested frequency, dosage and advice on how to optimize neuromuscular development and suggested the need to narrow the age range of the boys in the study from six to 11.^29^, ^30^, ^31^ The health‐care professionals recommended exercises focusing on strength, balance, proprioception, flexibility and mobility. Hill et al's^32^ findings concur with the exercises identified by the health‐care professionals in our study and indicated that an important feature of their home‐based programme was the selection of balance, strengthening and flexibility exercises to maintain function and muscle performance. Takken et al^33^ identified the need to include similar components for their 12‐week home‐based exercise programme for children with chronic conditions and cancer, which focused on muscle strength, exercise capacity and functional mobility. Takken and colleagues’ study involved children undertaking exercises four times a week (twice at home and twice in clinic) with children's ages ranging from six to fourteen. Yet, the programme was reported to be too demanding with only four out of 16 children completing the entire programme, as well as the ages of the children being too broad as they observed that the younger and older children were challenged and motivated by different exercises. When designing and developing the intervention in our study, the health‐care professionals indicated that practically and clinically the dosage should be twice a week, with a narrower age range to account for neuromuscular maturation and development.^33^

Through our study, we found that having a home‐based setting with regular one‐to‐one physiotherapy support would facilitate and promote greater compliance and adherence.^34^ In a study by Lillo‐Navarro et al^35^ on a home‐based exercise programme for children with physical disabilities, the parents perceived that their children's adherence to the programme was more successful taking into account the physiotherapist's teaching style. The findings suggested that the parents appreciated professional suggestions for incorporating home‐based exercises into their daily routine in order to overcome the challenges of adherence. The findings of our study concur with Lillo‐Navarro and colleagues’ and provide vital experiential knowledge that may be used by health‐care professionals to consider tasks and strategies that may be used to develop and implement a home exercise programme with adequate levels of adherence.^35^

There was a degree of uncertainly whether incentivization induced regular adherence. Incentivization in the form of payment may not affect significantly on improving compliance. Takken et al^33^ suggested that when children have fun during exercises, compliance might be better. They indicate that the psycho‐social component is important for both parents and children.^33^ Parental motivation such as adapting family routines and making exercise a family activity and seeing benefit increases adherence to exercise.^36^ In Birt et al's study^36^ on home‐based physiotherapy treatment in children and young people with joint hypermobility, they found that non‐adherence was associated with lower levels of parental supervision, not understanding the treatment, not seeing benefit and not having specific time dedicated to doing the exercises. Parents’ role in motivating and managing children to undertake the exercises is central to adherence.^36^

The study's physiotherapists made a crucial observation about taking the study forward. They suggested developing a set of online tools to support and aid parents to deliver the intervention. Wagner et al's^37^ study on establishing an online physical exercise programme for people with haemophilia suggests that online exercise instructions offer individually adapted exercise information for regular free home‐based training in order to benefit from increased physical fitness and joint stability.^37^ In a study by Alderdice et al^38^ on parents caring for preterm infants, they found that parents were generally positive in using websites for information and support; yet, finding relevant evidence‐based information was challenging. Therefore, it is important to understand the information and support needs of parents to be able to obtain high‐quality, evidence‐based resources online which are easily accessible, easy‐to‐understand, trustworthy and parent‐centred.^38^

### 5.1 Study Limitations

The findings from this study relate to a patient group with a specific rare disease, and it is acknowledged that their experiences may not be comparable to other conditions or disease groups. The literature review was undertaken to present the gaps in evidence to the health‐care professionals involved in the modified NGT activity (Study 1), rather than as a full systematic literature review, and it is therefore acknowledged that this has methodological limitations as only titles and abstracts were reviewed, rather than a full review of papers being conducted. As the participants in Study 1 were recruited via the UK Haemophilia Society, this may have created a bias around the boys and parents (almost all mothers) who may have positive views around exercise and physical activity. The eventual numbers included in the study may seem small, which is largely explained by the rare occurrence of haemophilia; however, it is expected that by progressing to a larger trial, recruitment across 11 to 12 study sites will increase recruitment pools.

## 6 CONCLUSION

The DOLPHIN study drew upon the person‐based approach to involve boys, families and health‐care professionals to design, develop and refine a muscle strengthening exercise programme for boys with haemophilia. A new intervention was designed, which was then demonstrated and refined through engagement with the boys and their families. The boys, families and the study's physiotherapists were consulted following its delivery to evaluate any elements of the programme that did not work. The intervention was generally acceptable to the patients with some refinements necessary prior to progressing to a future RCT. In addition, it has been shown how involving users can potentially help researchers to simply and effectively address any challenges posed when working with children with rare diseases.

## CONFLICT OF INTEREST

The authors declare no conflicts of interest.

## AUTHOR CONTRIBUTION

FH involved in the study design, conduct and analysis of the qualitative data. FH, DS, WD, MB and TPH conceived the original study. FH attained University of Kent ethical approval for Study 1, and DS obtained NHS ethical approval for Study 2. FH prepared the initial draft of the paper with input from DS. DS, WD, MB, LC, TPH and ES provided critical review of the manuscript.

## ETHICAL APPROVAL

For Study 1, ethical approval was granted by the University of Kent School of Social Policy, Sociology and Social Research—SRC Research Ethics Committee (SRCEA id 177). For Study 2, ethical approval undertaken by the NHS Research Ethics Committee was granted by the Health Research Authority and London—Fulham Research Ethics Committee (17/LO/2043).

## Data Availability

The datasets generated and analysed during the current study are not publicly available due to privacy and ethical reasons but are available from the corresponding author on reasonable request.
